# Effect of Oligosaccharide Degree of Polymerization on the Induction of Xylan-Degrading Enzymes by *Fusarium oxysporum *f. sp.* Lycopersici*

**DOI:** 10.3390/molecules25245849

**Published:** 2020-12-11

**Authors:** Nasim Najjarzadeh, Leonidas Matsakas, Ulrika Rova, Paul Christakopoulos

**Affiliations:** Biochemical Process Engineering, Division of Chemical Engineering, Department of Civil, Environmental and Natural Resources Engineering, Luleå University of Technology, 97187 Luleå, Sweden; nasim.najjarzadeh@ltu.se (N.N.); leonidas.matsakas@ltu.se (L.M.); ulrika.rova@ltu.se (U.R.)

**Keywords:** xylooligosaccharides, xylanases, induction, *Fusarium oxysporum*, enzymatic assay

## Abstract

Xylan is one of the most abundant carbohydrates on Earth. Complete degradation of xylan is achieved by the collaborative action of endo-β-1,4-xylanases and β-d-xylosidases and a number of accessories enzymes. In filamentous fungi, the xylanolytic system is controlled through induction and repression. However, the exact mechanism remains unclear. Substrates containing xylan promote the induction of xylanases, which release xylooligosaccharides. These, in turn, induce expression of xylanase-encoding genes. Here, we aimed to determine which xylan degradation products acted as inducers, and whether the size of the released oligomer correlated with its induction strength. To this end, we compared xylanase production by different inducers, such as sophorose, lactose, cellooligosaccharides, and xylooligosaccharides in *Fusarium oxysporum *f. sp.* lycopersici*. Results indicate that xylooligosaccharides are more effective than other substrates at inducing endoxylanase and β-xylosidases. Moreover, we report a correlation between the degree of xylooligosaccharide polymerization and induction efficiency of each enzyme. Specifically, xylotetraose is the best inducer of endoxylanase, xylohexaose of extracellular β-xylosidase, and xylobiose of cell-bound β-xylosidase.

## 1. Introduction

Xylan forms part of hemicellulose and constitutes nearly 1/3 of plant biomass, making it one of the most abundant carbohydrates on the planet [1]. Hemicellulose, cellulose, and lignin represent the three main components of the plant cell wall. They are bound to each other by covalent and non-covalent bonds, which provide plants with cell wall integrity and fiber cohesion [2]. Xylan is linked to other polysaccharides through hydrogen bonds and to ferulic and coumaric acid units of lignin through covalent linkage of arabinofuranosyl side chains [3]. It is located mainly in secondary cell walls [4] of hardwoods, softwoods, and annual plants [5]. In hardwoods, hemicellulose consists mostly of glucuronoxylan, whereas in softwoods, it comprises mainly glucomannan and to a smaller extent glucuronoxylan. Depending on the plant species, its structure and composition can differ significantly [6].

Although xylans with a linear unsubstituted structure can be found in esparto grass [7], tobacco [8], and some marine algae [9], they are found mainly as heteropolysaccharides with a backbone of 1,4-linked β-d-xylopyranosyl units and different side groups, including glucuronopyranosyl, 4-*O*-methyl-d-glucuronopyranosyl, α-l-arabinofuranosyl, acetyl, feruloyl, and/or p-coumaroyl. In hardwoods, xylan is found mostly as *O*-acetyl-4-*O*-methylglucuronoxylan, while in softwoods and annual plants it is in the form of arabino-4-*O*-methylglucuronoxylan and arabinoglucuronoxylan, respectively [10]. Several enzymes with different specific activities are required to completely break up such complex polymers.

Xylanases represent a group of glycosyl-hydrolases (GHs) responsible for the hydrolysis of the xylan backbone. Fungal xylanases belong mainly to CAZy GH families 10 and 11, with the former releasing products with lower molecular weight [3]. Xylanases include mainly endo-β-1,4-xylanases (EC 3.2.1.8) and β-d-xylosidases (EC 3.2.1.37), as well as a recently reported group of GH30 enzymes exhibiting xylobiohydrolase activity [11]. To achieve better xylan degradation, endoxylanases rely on the synergistic action of side-chain removing enzymes [3,12,13], with other ones being required depending on xylan chain length, presence of side-chain substituents, and degree of branching [10,14,15]. Endo-β-1,4-xylanase hydrolyzes the glycosidic bond of xylan and produces xylooligosaccharides, which are subsequently hydrolyzed by β-xylosidase [16]. Endoxylanases are sensitive to the presence of adjacent substituents, linkage type, degree of xylan branching, and type of branched sugar [17]. β-xylosidases are categorized in 11 GH families, with fungal β-xylosidases belonging to GH3 and GH43 CAZy families [10]. They release xylose either by cleaving xylobiose or by attacking the non-reducing end of short-chain xylooligosaccharides to generate monomers [18,19]. This step is crucial to prevent end-product inhibition of endoxylanases by accumulated xylobiose [10].

Xylanases are of increasing importance in food, wastewater treatment, single-cell proteins production, biofuel, pulp, and paper industries [2,6]. One of their main applications is as a starting material for the production of xylitol from wood residue [6].

Xylanases are produced by a variety of organisms, such as marine algae, protozoa, crustaceans, insects, snails, seeds of land plants, bacteria, and filamentous fungi, actinomycetes, and yeasts [20,21,22,23]. Filamentous fungi secrete large amounts of biomass-degrading enzymes including xylanases [19]. The genera *Trichoderma*, *Aspergillus*, *Fusarium*, and *Thermomyces* are good candidates for industrial applications [24,25]. *Fusarium oxysporum* is a plant pathogen that causes significant economic loss due to its ability to infect and induce rotting of the roots of more than 100 plant species [26]. Not surprisingly, this species possesses a wide collection of plant cell wall-degrading enzymes, many of which are secreted. From an industrial point of view, *F. oxysporum* is of great interest due to its ability to hydrolyze both cellulose [27] and hemicellulose [28], against which it has evolved at least four different xylanases [29], converting the resulting sugars to ethanol in a single consolidated step [30,31]. Moreover, one of the most important genera able to develop diseases in plants is *Fusarium* which not only produces losses by the fungal presence but also mycotoxin production harmful to human and animal consumers [32] and xylanases have been shown to play an important role during pathogenesis [33]. Understanding of the plant-pathogen interaction at enzyme induction levels would help in developing strategies to combat *Fusarium* disease.

The xylanolytic system in filamentous fungi responds to induction and repression [6,34]. Complex xylan-containing substrates, such as wheat bran [35] and corn cobs [36], are good inducers of xylanases. These enzymes release xylooligosaccharides, which are then transported inside the cells of microorganisms, where they are further broken down by intracellular xylanases. The final products are believed to induce xylanase-producing genes [37,38]. However, it remains to be determined which of the released oligomers behaves as an inducer, and if there is any relationship between oligomer size and induction efficiency. Addition of glucose, xylose, sucrose, fructose, and xylan to *Trichoderma asperellum* grown on oil palm empty fruit bunches significantly decreased xylanase production through catabolic repression of xylanase genes, with monosaccharides exhibiting the strongest repressive effect and xylan the weakest [39]. The same study found also that sucrose repressed the xylanase genes more effectively than cellobiose, which could be explained by its easier degradation to glucose and fructose. These results contradict a study on *Aspergillus niger* B03, in which xylan acted as an inducer [40,41]. Because xylose and xylan serve as repressors in filamentous fungi, it is likely that the derivatives of xylan hydrolysis (e.g., xylobiose, xylotriose, and other xylooligosaccharides) act as inducers. Other studies have shown that xylan in *F. oxysporum* and xylose and arabinose in *A. niger* are inducers of xylan-degrading enzymes [42,43]. Xylose and lactose are known to be potential inducers in some microorganisms, such as *Trichoderma longibrachiatum* and *Aspergillus fischeri* [44,45], but are ineffective in *F. oxysporum* [36]. Two low-molecular weight endoxylanases (I and II) isolated from *F. oxysporum* F3 were found capable of binding to crystalline cellulose but not insoluble xylan [46]. All of these results suggest a complex induction and repression mechanism of xylanases.

Such complexity can be attributed to the different physiological and metabolic properties of microorganisms, fermentation type (solid state or submerged fermentation), composition of culture medium, and carbon source (pure sugars or lignocellulosic substrates) [6,39]. In most instances where xylose and xylan induced xylanases, these substrates were added as pure carbon sources in submerged fermentation. However, in media composed of lignocellulosic substrates, the addition of easily-metabolized sugars at concentrations above 2% and 3% *w*/*w* represses these genes [24,47]. Hence, the concentration of the compound also dictates whether it acts as inducer or not. This was confirmed in *A. niger*, whereby a low concentration of d-xylose induced xylanases, whereas a higher one repressed them through CreA [48]. CreA is a C2H2 finger domain that functions as a transcription repressor of CAZymes in filamentous fungi, such as *A. niger* and *Aspergillus nidulans* [49,50]. Cre1 is its ortholog and has similar function in *Neurospora crassa* and *Trichoderma ressei* [49,51,52]. Stability and function of CreA depend on the CreB–CreC deubiquitination complex, which contributes to the carbon catabolite repression of CreA [49,53,54,55].

In this study, we aimed to elucidate the mechanism underlying induction of xylan-degrading enzymes in response to different inducers, such as xylooligosaccharides, cellooligosaccharides, sophorose, and lactose. Applying inducers with different degrees of polymerization and concentration allowed us to determine a correlation between these factors and their induction strength.

## 2. Results

### 2.1. Effect of Inducers on Enzyme Activity

#### 2.1.1. Endo-β-1,4-Xylanase

**Figure 1:**
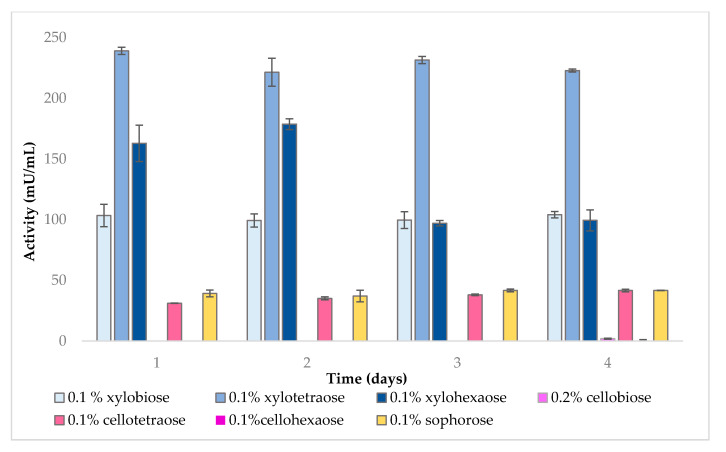
Endo-β-1,4-xylanase activity in the presence of different inducers.

Endo-β-1,4-xylanase activity was measured in the presence of different inducers over a period of 4 days. As shown in Figure 1, xylooligosaccharides were more efficient than other types of sugars at inducing endo-β-1,4-xylanase activity. Maximum induction was achieved with xylotetraose, followed by xylohexaose and xylobiose.

While no activity was detected upon addition of lactose, control and cellobiose, endo-β-1,4-xylanase was induced to a lesser extent by sophorose and cellotetraose. Interestingly, 0.1% cellotetraose led to considerably higher enzyme induction compared to 0.2% cellobiose or 0.1% cellohexaose. This result is in contrast with a previous study, whereby 1% (*w*/*v*) cellobiose and cellulose could induce xylanase in *F. oxysporum* [36], but can be explained by the much lower concentration of cellobiose used here.

#### 2.1.2. Extracellular β-Xylosidase

**Figure 2:**
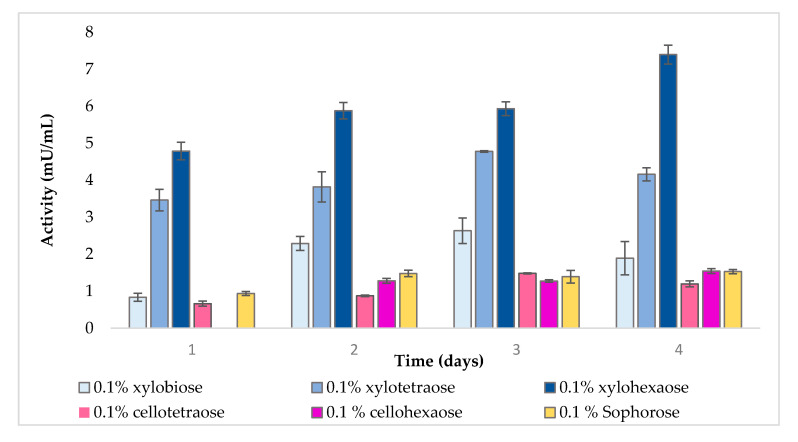
Extracellular β-xylosidase activity in the presence of different inducers.

As shown in Figure 2, xylooligomers appeared to be considerably better inducers of extracellular β-xylosidase compared to other compounds. Moreover, there was a positive correlation between the length of the inducer and its induction strength. Hence, xylohexaose was the best inducer, followed by xylotetraose and xylobiose. Sophorose, cellotetraose, and cellohexaose achieved similar low extracellular β-xylosidase induction, whereas the control showed no activity.

#### 2.1.3. Cell-Bound β-Xylosidase

**Figure 3:**
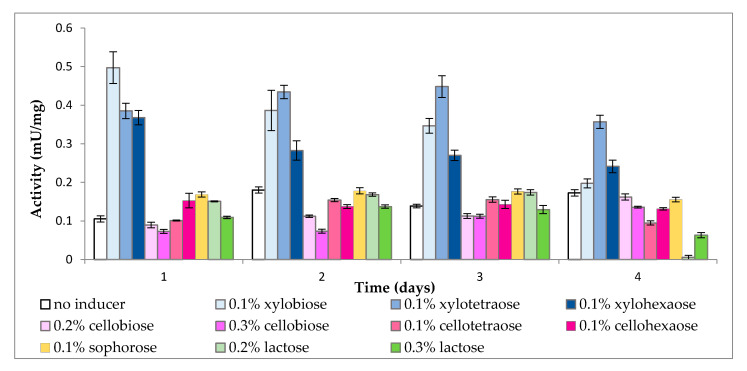
Cell-bound β-xylosidase activity in the presence of different inducers.

As is evident from Figure 3, all samples, even the control, demonstrated some cell-bound β-xylosidase activity, which could be ascribed to the presence of transporters with β-1,4-cleaving capacity. Again, xylooligomers were the most effective inducers, while the remaining sugars failed to increase enzyme activity beyond that of the control. However, the length of xylooligomers had an inverse effect on cell wall-bound β-xylosidase activity, with xylobiose exerting stronger induction at the beginning and xylotetraose later on, while xylohexaose was generally weaker than the other two.

### 2.2. Uptake Experiment

#### 2.2.1. Concentration of Xylooligosaccharides in the Medium

**Figure 4:**
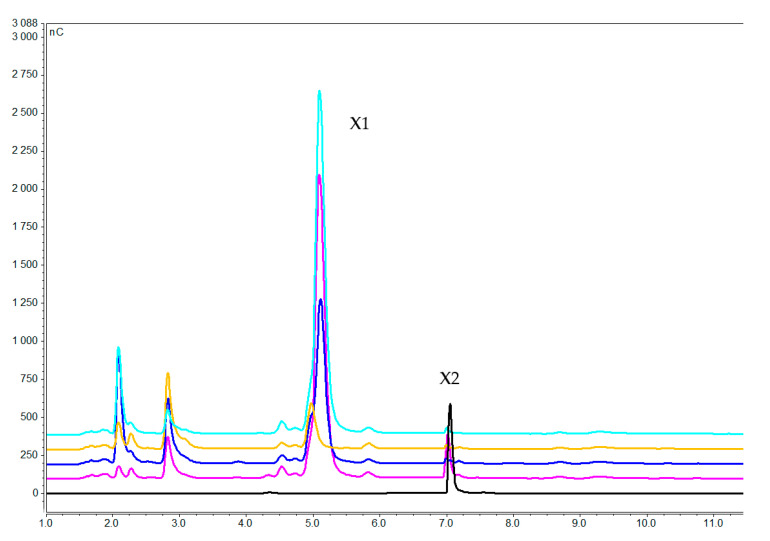
Xylooligosacharides concentration in the medium after 1 h (pink), 3 h (dark blue), and 7 h (orange) of induction with xylobiose. Blue and black lines correspond to pure xylose and xylobiose respectively (X1 = xylose, X2 = xylobiose).

**Figure 5:**
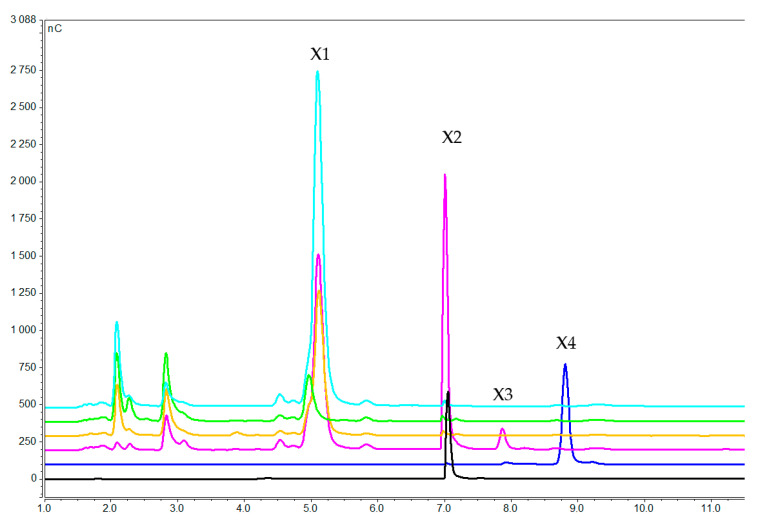
Xylooligosaccharides concentration in the medium after 1 h (pink), 3 h (orange), and 7 h (green) of induction with xylotetraose. Blue, black and dark blue lines correspond to pure xylose, xylobiose and xylotetraose, respectively (X1 = xylose, X2 = xylobiose, X3 = xylotriose, X4 = xylotetraose).

**Figure 6:**
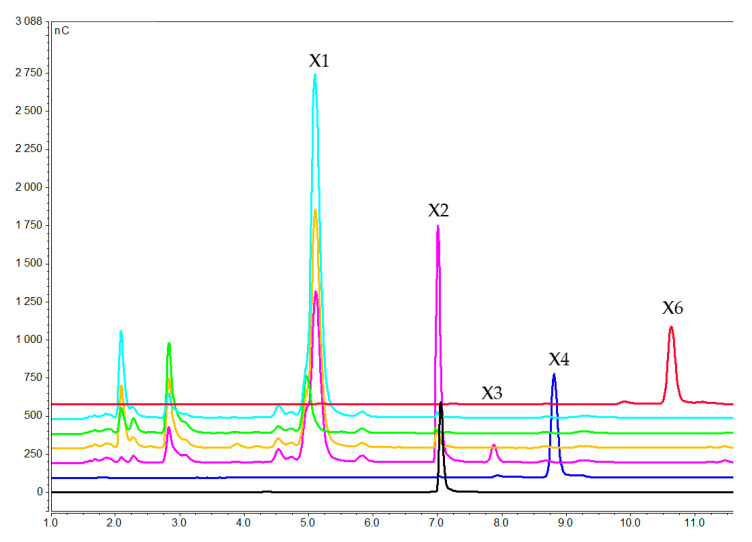
Xylooligosaccharides concentration in the medium after 1 h (pink), 3 h (orange), and 7 h (green) of induction with xylohexaose. Blue, black, dark blue and red lines correspond to pure xylose, xylobiose, xylotetraose, and xylohexaose respectively (X1 = xylose, X2 = xylobiose, X3 = xylotriose, X4 = xylotetraose; X6 = xylohexaose).

Analysis of the medium revealed degradation of xylooligosaccharides in all samples between 1 and 7 h. Xylobiose as inducer was nearly complete degraded to xylose after 3 h (Figure 4); whereas xylotetraose and xylohexaose were apparently broken up immediately as they could not be detected after as little as 1 h (Figure 5 and Figure 6) generating mainly xylose, xylobiose and xylotriose. This is in agreement with previous reports that *F. oxysporum* xylanolytic system has an endo character and clearly attacks mainly the internal glycosidic bonds, releasing xylobiose and xylotriose [46,56].

#### 2.2.2. Intracellular Concentration of Xylooligosaccharides

**Figure 7:**
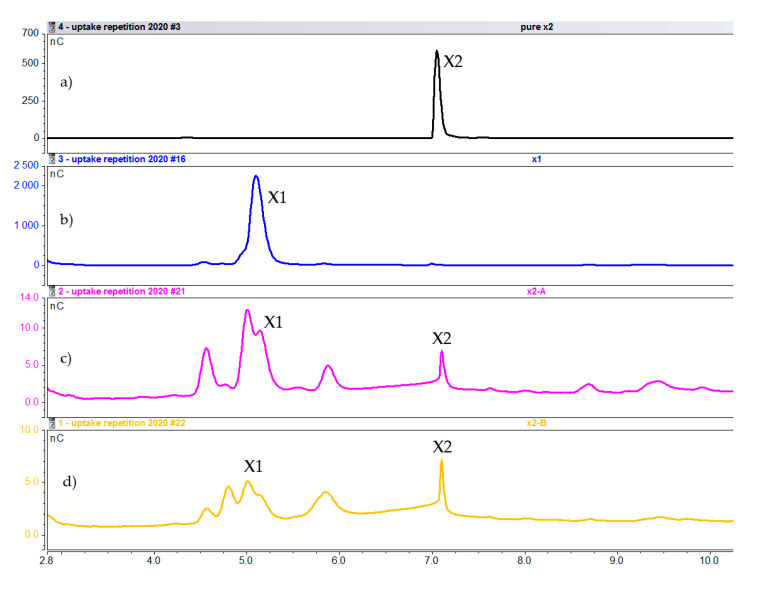
Intracellular composition after 1 h of induction with xylobiose. (**a**) Pure xylobiose. (**b**) Pure xylose (**c**) Cell extract fraction A. (**d**) Cell extract fraction B (X1 = xylose, X2 = xylobiose).

**Figure 8:**
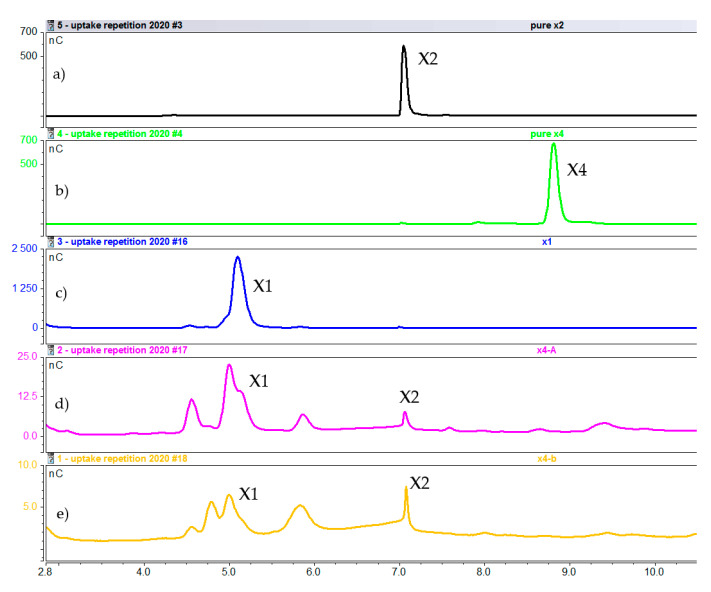
Intracellular composition after 1 h of induction with xylotetraose. (**a**) Pure xylobiose. (**b**) Pure xylotetraose. (**c**) Pure xylose (**d**) Cell extract fraction A. (**e**) Cell extract fraction B (X1 = xylose; X2 = xylobiose, X4 = xylotetraose).

**Figure 9:**
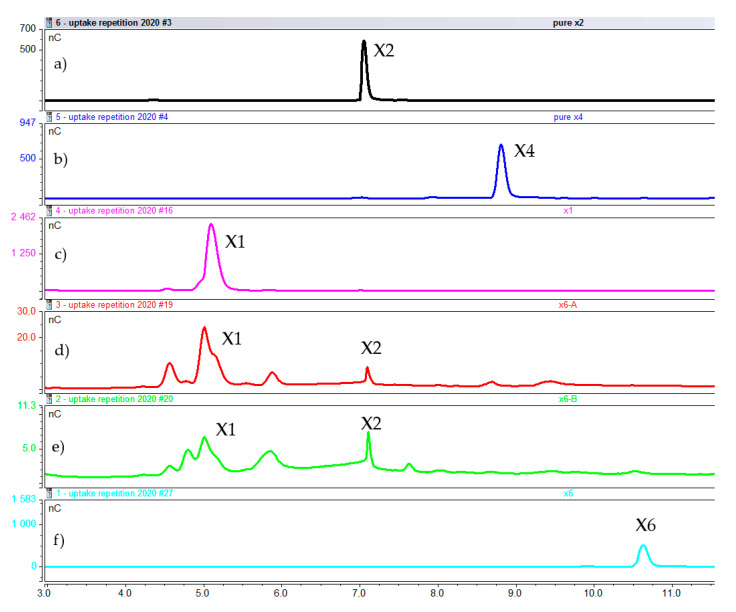
Intracellular composition after 1 h of induction with xylohexaose. (**a**) Pure xylobiose. (**b**) Pure xylotetraose. (**c**) Pure xylose (**d**) Cell extract fraction A. (**e**) Cell extract fraction B (**f**) pure xylohexaose (X1 = xylose, X2 = xylobiose, X4 = xylotetraose, X6 = xylohexaose).

Extracts of samples induced by xylobiose, xylotetraose, and xylohexaose revealed the presence of only xylobiose in cellular fractions A and B (Figure 7, Figure 8 and Figure 9), which are the cell extracts after 10 min and 4 h of boiling. This finding suggests that xylobiose was the most probable xylooligosaccharide degradation product to enter the cell and induce expression of genes related to xylan break up.

## 3. Discussion

Substrates containing easily metabolized carbon sources, such as xylose, lactose, sophorose, and xylan are excellent candidates for industrial production of xylanases. Xylose and xylan [6,40], as well as simple sugars derived from lignocellulosic substrates are effective xylanase inducers. While a mixture of these sugars was suggested to potentially improve production of xylanases [39], it remains unclear how a microorganism reacts to each individual inducer [24,39,47]. Here, we evaluated the effect of different xylooligosaccharides and cellooligosaccharides on induction of xylanolytic enzymes by *F. oxysporum*. Moreover, their effect was compared to sophorose, which is the most powerful xylanase inducer, and lactose, which is employed for industrial production of hydrolytic enzymes [57,58,59]. Furthermore, due to the complicated process of induction, the process was simplified by using a linear substrate rather than hemicellulosic substrates with side-chains to focus on the correlation between degree of polymerization and the induction power of inducers.

Generally, enzyme production is a reflection of gene regulation. Constitutive xylanases degrade xylan and produce xylose, xylobiose, xylotriose, as well as other oligosaccharides [60,61], which can further induce xylanase production. Unlike xylose, which can easily enter microbial cells [40,62,63], larger products require more energy to be transported [61,64]. This can occur via the release of xylooligomers by xylanases, followed by transport to the cell matrix and degradation by intracellular β-xylosidase to produce xylose [65,66]. Alternatively, transporters with β-1,4-cleaving activity hydrolyze oligomers to monomers during transport from the cell wall to the cell matrix [40].

Present results provide novel insights into the link between length of the inducer and induction of sugar-degrading enzymes. Accordingly, xylotetraose appeared to be a better endoxylanase inducer than xylohexaose or xylobiose. As the latter contains only two xylose subunits, the cell needs to secrete less endoxylanase or β-xylosidase compared to xylotetraose or xylohexaose, and can instead guide the energy toward producing more cell-bound β-xylosidase.

The uptake results suggest that xylobiose is the main product of endoxylanase-mediated degradation and its intracellular detection indicates that xylobiose is the imported saccharide into the cell. Thus, the synergistic action of endoxylanase and β-xylosidase leads to xylobiose release and transportation into the cell.

Long-chain xylooligosaccharides such as xylohexaose cannot be transported easily into the cell, and need to be first broken up in smaller residues. Compared to xylotetraose and particularly xylobiose, xylohexaose is broken up into more residues and for that requires more extracellular β-glucosidase to convert xylotriose to xylobiose. Therefore, xylohexaose induces the highest amount of extracellular β-glucosidase, followed by xylotetraose and xylobiose.

The key step in biomass degradation is appropriate sensing of substrate in the medium, which then triggers the enzymatic induction cascade [67]. Transporters play an important role in this context by sensing and taking up soluble sugars inside the cell, as well as by enabling communication between the cell and the surrounding environment [67]. Transporters are known to influence CAZymes-encoding genes by carrying small inducers from the environment into the cell [67,68,69,70]. The major facilitator superfamily (MFS) and ATP binding cassette (ABC) transporters are the most studied examples in fungi [71,72,73].

ABC transporters make up the largest family of membrane transport proteins. They are actively involved in the transport of heavy metals, sugars, lipids, drugs, and secondary metabolites, cell signaling, as well as resistance to toxic compounds [74,75,76]. The ability to export toxic chemicals out of the cell allows fungi to fend off toxins in the soil or plant defense compound [74]. ABC transporters comprise 45 known families [77] found mainly in prokaryotes, with some of them acting also as sugar transporters [73,78].

The genome of filamentous fungi encodes numerous MFS transporters [79,80] divided across 17 families. Comprising of a single polypeptide, families 1, 5, and 7 transport sugars, such as pentoses and hexoses inside the cell [81,82,83] through a mechanism that uses free energy obtained from a chemiosmotic ion gradient [77,84]. MFS transporters translocate also nucleosides, lipids, amino acids, peptides, and ions [84]. The main feature of MFS transporters in filamentous fungi is their ability to sense and carry more than one kind of sugar [67]. The type of carbon source controls transcriptional regulation of sugar transporters in fungi [74,85,86]. For example, the MFS family CDT2 hexose transporter in *N. crassa* [73,87] collaborates with the GH43-2/7 family of CAZymes to promote xylobiose, xylotriose, and xylotetraose, consumption [73,88]. In our experiment, transporters carried xylobiose inside the cell to trigger expression of xylan-degrading enzymes. A similar result has been observed in *Aspergillus fumigatus* Z5, whereby xylan induced endoxylanases and β-xylosidases to produce xylobiose, while specific sugar transporters translocated it into the cell [68,89]. There, it was further broken down to xylose through induction of intracellular β-xylosidases [90].

In summary, the present results have identified the products of xylooligomer degradation responsible for induction of xylanolytic enzymes in *F. oxysporum* and uncovered how their degree of polymerization affected the strength of induction.

## 4. Materials and Methods

### 4.1. Microorganism and Pre-Cultures

All chemicals were purchased from Sigma-Aldrich (St. Louis, MO, USA) unless otherwise stated. *F. oxysporum *f. sp.* lycopersici* (CBS 123668) was obtained from Centraal bureau voor Schimmelcultures (Utrecht, The Netherlands) and maintained on potato dextrose agar (39 g/L). The fungus was initially grown for 2 days in pre-culture medium containing yeast extract (20 g/L) and malt extract (5 g/L) at 29 °C in a shaking incubator (190 rpm).

### 4.2. Enzyme Induction Trials

To study the production of xylanolytic enzymes, *F. oxysporum* was first grown in medium containing 0.2 M sodium phosphate buffer, 0.3 g/L MgSO_4_·7H_2_O, 1 g/L KH_2_PO_4_, 10 g/L (NH_4_)_2_HPO_4_, and 1% *w*/*v* sucrose. The medium was inoculated with 6% *v*/*v* of pre-culture and incubated for 2 days in a shaking incubator. Cells were harvested through a sterile vacuum filter (0.45 µm, Sarstedt, Nümbrecht, Germany), washed with sterile milliQ water, and transferred to the same medium as above, but without sucrose. Cells were incubated for 24 h to ensure that the mycelium was free of any residual sugars. Then, individual compounds with potential inducer activity were added at the following final concentrations: 0.1% *w*/*v* sophorose (Megazyme, Wicklow, Ireland); 0.2 and 0.3% *w*/*v* cellobiose; 0.2 and 0.3% *w*/*v* lactose; 0.1% *w*/*v* xylobiose, xylotetraose, and xylohexaose (Megazyme); and 0.1% *w*/*v* cellotetraose and cellohexaose (Megazyme). The fungal culture was incubated at 29 °C and 190 rpm. To minimize errors during sampling, each sample point consisted of a separate flask, whose entire content was harvested and filtered. The supernatant and biomass fractions were used to determine extracellular and cell-bound xylanase activity, respectively, as described below.

### 4.3. Enzyme Activity Assessment

The cell filtrate (supernatant) was used to determine extracellular endoxylanase and β-xylosidase enzyme activities. Specifically, for endo-β-1,4-xylanase activity, a solution of 1% *w*/*v* xylan from birch wood dissolved in phosphate-citrate buffer (pH 5) was used as substrate and 200 µL of this solution was incubated with 50 µL enzyme at 40 °C for 30 min. The released reducing sugars were measured by the dinitrosalicylic acid method, using a xylose standard curve [91]. For β-xylosidase activity determination, 1 mM p-nitrophenyl-β-d-xylopyranoside was used as substrate and 900 µL of this solution was incubated with 100 µL of the enzyme at 40 °C for 30 min. After the reaction was stopped by adding 200 mL 30% Na_2_CO_3_, the released p-nitrophenol was measured at 410 nm and compared to a standard curve prepared under similar conditions [28]. One unit (U) was defined as the amount of enzyme that released one μmole of product (glucose equivalent or p-nitrophenol) per minute from each substrate.

Cell-bound β-xylosidase activity was assessed following the same procedure as described above for extracellular β-xylosidase, with the difference that 1.5 mg of air-dried biomass was used as substrate. Enzyme activity was expressed in mU/mL and mU/mg for extracellular and cell-bound enzymes, respectively.

### 4.4. Xylooligosacharides Uptake Experiment

Xylooligosaccharides in the medium were separated by high-performance anion-exchange chromatography (HPAEC), after 1, 3, and 7 h of induction with different oligosaccharides. Upon repeating the experiment, the culture filtrates were analyzed by Dionex ICS-5000 system (Thermo Scientific, Waltham, MA USA) with a pulsed amperometric detector equipped with a disposable electrochemical gold electrode, using a CarboPac PA1 4 × 250 mm analytical column and a CarboPac PA1 4 × 50 mm guard column, at 30 °C.

The presence of xylooligosaccharides inside the cells after 1 h of induction with different oligosaccharides was measured by the same system. The culture was vacuum-filtered, and the towel-dried biomass was ground immediately in liquid nitrogen, washed with 5 mL water, boiled for 10 min, and centrifuged. The supernatant was aspirated to a separate tube and labeled as fraction A. Separately, 3 mL water was added to the precipitated biomass, which was then boiled for 4 h and the ensuing supernatant was labeled as fraction B.
